# 

**Published:** 1867

**Authors:** 


### THE CHICAGO MEDICAL JOURNAL.

**Edited by J. ADAMS ALLEN, M.D., LL.D.**
Prof. Principles and Practice of Medicine and Clinical Medicine in Rush Med. College.
All letters for the JOURNAL should be addressed to DR. J. ADAMS ALLEN, P.O. Box 1948, Chicago, Ill.

**TERMS---$2 PER ANNUM, IN ADVANCE.**

$3 if not paid before 1st of July in each year. Single copies, 25 cents.

BOOKS RECEIVED.

*Aitken's Science and Practice of Medicine*, with additions, By Meredith Clymer, M.D. Second volume. Philadelphia: LINDSAY & BLAKISTON. 1867.

*Obstetrics;* the Science and the Art. By Charles D. Meigs, M.D., lately Professor of Midwifery and Diseases of Women and Children, in Jefferson Medical College, of Philadelphia, etc., etc., etc. Fifth edition, revised, with one hundred and thirty illustratious. Philadelphia: HENRY C. LEA. 1867.

*Practical Dissections.* By Richard M. Hodges, M.D., formerly Demonstrator of Anatomy in the Medical Department of Harvard University. Second edition, thoroughly revised. Philadelphia: HENRY C. LEA, 1867. pp. 285.

*Inhalations* in the Treatment of Diseases of the Respiratory Passages, particularly as affected by the use of atomized fluids. By J. M. DaCosta, M.D., Physician to Pennsylvania Hospital; Fellow of College of Physicians and Surgeons, etc., etc. Philadelphia: J. B. LIPPINCOTT & Co. 1867.

*Injuries of the Spine;* with an Analysis of nearly four hundred cases. By JOHN ASHHURST, Jr., A.M., M.D., Fellow of College of Physicians of Philadelphia, etc., etc., etc. Philadelphia: J. B. LIPPINCOTT & Co. 1867. pp. 127.

*Code of Medical Ethics*, adopted by the American Medical Association. New York: WM. WOOD & Co. 1867.

*The Indigestions;* or Diseases of the Digestive Organs Functionally Treated. By THOMAS KING CHAMBERS, Honorary Physician to H. R. H. the Prince of Wales, Consulting Physician and Lecturer on the Practice of Medicine at St. Mary's Hospital, Consulting Physician to the Lock Hospital, Author of "Lectures Chiefly Clinical," etc. Philadelphia: HENRY C. LEA. 1867. Chicago: S. C. GRIGGS & Co. One volume, octavo, pp. 287.

*Why Not?* A book for every woman. The prize essay to which the American Medical Association awarded the gold medal for MDCCCLXV. By HORATIO ROBINSON STORER, M.D., of Boston, Professor of Obstetrics, etc., etc.

Issued for general circulation by order of the American Medical Association.

*Casta placent superis. Casta cum mente venito,*
*Et manibus puris sumita fontis aquam.*

Boston: Lea & Shepard. 1867. Pp. 99. From the author.

*Contributions to the Pathology, Diagnosis and Treatment of Angular Curvature of the Spine.* By BENJAMIN LEE, M.D., Philadelphia: LIPPINCOTT & Co. 1867. Pp. 129. From the Publishers.

*An Inquiry into the Origin of Modern Anæsthesia.* By the Hon. TRUMAN SMITH, M.C., etc., etc. Hartford: Brown & Gross. 1867. Pp. 165.

*On the Action of Medicines in the System.* By FREDERICK WILLIAM HEADLAND, M.D., B.A., F.L.S., Fellow of the Royal College of Physicians, etc., etc. Fifth American, from the Fourth London edition. Revised and enlarged. Philadelphia: LINDSAY & BLAKISTON. 1867. Pp. 431.

*Watson Abridged:* A Synopsis of the Lectures on the Principles and Practice of Physic. Delivered at King's College, London, by THOMAS WATSON, M.D., F.R.C.R., etc., etc. (Abridged from the last English edition.)

With a concise but complete account of the Properties, Uses, Preparations, Doses, etc. (Taken from the U. S. Dispensatory,) of all the medicines mentioned in these lectures, and with other valuable editions. By J. J. MEYLOR, A.M., M.D. Philadelphia: Published by the Author. 1867. Pp. 277. From the Author.

*Oesophagotomy*, for the Removal of Foreign Bodies. Pamphlet By DAVID W. CHEEVER, M.D., etc. Boston: DAVID CLAPP & SON, Printers. 1867. Pp. 46.

*Diphtheria.* A Prize Essay. By E. S. GAILLARD, M.D., Richmond, Va. Pp. 114. From the author.

*Half Yearly Abstract*, Rankin's. From HENRY C. LEA. Vol. 44. July to December, 1866.

*The Riverside Magazine for Young People.* The April number is one of unusual excellence, comprising great variety in its articles and illustrations, and is characterized by the prominence given to "out-door life." HURD & HOUGHTON, publishers, at 459 Broome Street, New York. Subscription price $2.50 per annum.

*Atlantic Monthly*, for May.

*Our Young Folks*, for May.

All the above for sale at the houses of W. B. KEEN & Co., and S. C. GRIGGS & Co., but received too late for further notice the present month.

